# Supplementary material for: Living the ‘Best Life’ or ‘One Size Fits All’—Stakeholder Perceptions of Racehorse Welfare
Source: Animals (Basel). 2019 Mar 31;9(4):134. doi: 10.3390/ani9040134 (PMC6523604; doi:10.3390/ani9040134)
Supplement: Supplementary file 1 [file animals-09-00134-s001.pdf]

animals

MDPI

Article

Supplementary Materials: Living the 'Best Life' or 'One Size Fits All'—Stakeholder Perceptions of Racehorse Welfare

Deborah Butler *, Mathilde Valenchon, Rachel Annan, Helen R. Whay and Siobhan Mullan

School of Veterinary Sciences, University of Bristol, Langford, Somerset BS40 5DU, UK; mathilde.valenchon@bristol.ac.uk (M.V.); rachel.annan@bristol.ac.uk (R.A.); Bec.Whay@bristol.ac.uk (H.R.W.); Siobhan.Mullan@bristol.ac.uk (S.M.)

* Correspondence: deborah.butler@bristol.ac.uk

Received: 6 March 2019; Accepted: 28 March 2019; Published: date

Table S1. Stakeholders perceptions of minimum welfare standards and the best 'life' a horse in training can live for health (58 statements, ranked 1st).

Minimum welfare standards (24 statements in total)	'Best life' (34 statements in total)
**Veterinary care (9 statements)**	**Veterinary care (12 statements)**
Appropriate vet support	Immediate vet support
Welfare of horses' paramount, vet, essential for injury, disease	Constant veterinary care
Lameness investigated and treated if lame	Welfare improved through an improvement in vet care/diagnostics
Vaccination as required- reasonable	Vaccination, reasonable, risk based
Ulcers under grade3/4 for 'acceptable' welfare	No ulcers
Stomach, yearly gastroscopy.	Stomach, twice yearly gastroscopy
Treatment. Always following an attempt at a diagnosis. Evidence based treatment	Treatment. Always following an attempt at diagnosis.
Vet care basic and only when needed	Vet in the yard
Vet in emergency, vet care when needed	Vet immediately available
	Evidence based treatment not anecdotal
	Vets on hand
	Wind only treated in response to a problem
**Health care (4 statements)**	**Health care (9 statements)**
(Basic) free from pain	Team work (farrier, vet, physio, dentist etc)
General health check	Collaborative preventative health care for individual horses
Dentistry once yearly by a qualified equine dentistry technician or a vet	Dentistry twice yearly by a qualified equine dentistry technician or a vet
Disease control, keep alive!	Sound in all gaits (happy athlete)
	Individuals in terms of health care
	Massage, teeth, worming
	Little and often work rather than concentrated pressure to keep healthy
	Disease control, quarantine sick horse but ideally in view of other horses
	Bred to sound and healthy

Veterinary interventions (3 statements)	Veterinary interventions (4 statements)
Breathing in general, treatment if needed<br>Overuse of medication to keep a horse racing<br>Unnecessary use of wind operations	Breathing, no respiratory system disease<br>Wind only treated in response to a problem<br>Excellent veterinary treatment when needed at races<br>Use of treatment /paste for gastric ulcers
**Farriery (8 statements)**	**Farriery (5 statements)**
Good shoeing<br>Basic farriery<br>Gd farrier but comes every 6 wks but only does what he is told<br>Farrier 6 wks or when lost a shoe<br>Shoes left in too long to cut costs<br>Poorly shod for conformation and stage of training, lazy farriers<br>Shoeing to soundness<br>Shoeing before a race and when needed	Farrier- basic and regular plus specialist if required<br>Regular shoeing from farrier who knows the horses<br>Farrier (foot balanced)<br>Shod appropriately to its discipline and stage of training<br>Farrier who knows the horse's feet
**Para-professionals (0)**	**Para-professionals (3 statements)**
	Chiropracter<br>Regular physio and chiro (massage etc)<br>Industry experts with no incentive to present to the vet
	**Additional therapies (1 statement)**
	Ice boots, salt box, hydro chamber, osteopath, sea walker, spa, solarium, swimming pool

Table S2. Stakeholders perceptions regarding minimum and best 'life' with regard to training, exercise and recovery (43 statements, ranked 2nd).

Minimum welfare standards (21 statements in total)	'Best life' (22 statements in total)
**Exercise (9 statements)**	**Exercise (11 statements)**
Fit horses leave their box every day<br>Only worked when sound<br>Ridden 6 days a week<br>Exercised 6 days/week<br>Exercised 6 days/week<br>Horse is fit enough to participate<br>Comfortable in canter<br>Walker every day and alternate direction every other day<br>Exercised daily	Qualified flatwork training (a well-balanced horse will suffer less injuries)<br>Cross training and varying working routine for horses mental and physical well being<br>More time riding out<br>Plenty of time to cool down after exercise<br>Exercise regime to suit individual horse<br>Ridden and warmed up, cooled down<br>Exercised daily<br>Exercise, how much, how often<br>Maintain condition, fitness<br>Daily exercise<br>Exercise about an hour, safe surfaces
**Training (1 statement)**	**Training (7 statements)**
At a min, horses should be used to different surfaces so don't struggle on grass after all-weather surface	Appropriate training and exercise to [be able] to ride safely<br>Race day experience is as stress free as possible

	after a considered preparation<br>Best surfaces<br>High quality gallop surfaces to reduce injuries<br>No queuing for gallops<br>Yards own gallops well maintained<br>Short breaks to recuperate where fitness is not lost
**Horse-human education (6 statements)**	**Horse-human education (1 statement)**
Education of horses re stalls, education at home<br>Preparation safe, schooled properly for horse and rider<br>Staff/riders do no harm<br>Minimum care after exercise as 'rider outers' used rather than 'paid' lads<br>Riders not regular<br>When rider outers don't know the horse and put back in the box without basic husbandry attended to like sponging off	Riders to be assigned to ride horses where they suited the horses individually, in terms of rider temperament
**Transport (1 statement)**	**Transport (2 statements)**
Even 6+ trip to races done in a day	Transport enough room, not too long a journey, educated to load properly, safe<br>Travelling, quiet, temp controlled, water/hay, adequate travelling staff. One person familiar with the horse
**Recovery (4 statements)**	**Recovery (1 statements)**
Too much racing<br>Just doing as little as possible after a run to let the horse recover after a run<br>Loaded up too quickly after a run<br>Lack of time to have a pick of grass after exercise	Travelling for horses (rested between meetings)

Table S3. Perceptions of participants' opinions regarding minimum and best 'life' with regard to physical comfort/living environment (40 statements, ranked 3rd).

Minimum welfare standards (16 statements in total)	'Best life' (24 statements in total)
**Bedding (5 statements)**	**Bedding (5 statements)**
Suitable stabling-clean bedding, maintenance routine<br>Dust free, adequate bedding<br>Minimal basic bedding<br>Bedding, not just mats<br>Limited shavings, safe, clean, dry, gd light, size enough to roll	Housing suited to individual's character and some in barns, some set apart, some closed in, some opened up<br>Deep straw bed and mucked out twice a day<br>Deep bed, rubber mats<br>Plenty of bedding<br>Padded walls, mats, plenty of bedding, shavings?
**Stabling (5 statements)**	**Stabling (5 statements)**
Relaxation time in the box limited<br>Wooden boxes<br>Min shelter from elements<br>Safe stable, enough bed so can't make contact with the floor	Large, light stable<br>Clean stabling, mucked out every day, clean bedding<br>Grills between stables, sight and smell<br>Environment to minimise stress<br>View- to prevent boredom, crib biting

Size of stable/stalls. Should be able to get up and down	
**Tack (2 statements)**	**Tack (6 statements)**
Correctly fitted tack<br>Adequate fitted tack	Tack supplemented with sheepskin pads and girth covers<br>Tack fitted and tailored to each horse<br>Own bridle with well fitting saddle<br>Dedicated tack with regular saddle checks<br>Well fitted tack with full tree saddle<br>Comfortable safe tack
**Ventilation (2 statements)**	**Ventilation (5 statements)**
Adequate daylight and ventilation<br>Airflow	Temp. Optimum controlled environment<br>Well ventilated stables<br>Ventilation. No outside access, never too hot/cold?<br>Good ventilation<br>Stable gd air flow, not damp, light
**Rugs (2 statements)**	**Rugs (3 statements)**
Rug for each weather condition<br>Rugged in winter	Warmth/cold, varying by season<br>Clipped in winter, rugged according to weather conditions<br>Used appropriately depending on the weather conditions

Table S4. Stakeholder perceptions regarding minimum and best 'life' with regard to feeding (30 statements, ranked 4th).

Minimum welfare standards (16 statements in total)	'Best life' (15 statements in total)
**Forage and hard food (6 statements)**	**Forage and hard food (10 statements)**
Good feed & man doing the feeding all the time, knows horses inside out<br>Safe food, not out of date<br>Concentrates and roughage.<br>Sufficient feed and haylage<br>Feed, liberal roughage, *ad-lib* hay, hard feed based on current exercise<br>Good quality clean forage and feed (not harmful)	Top quality feedstuffs and supplements<br>Feed, fresh, balanced, spread out over the day<br>*Ad-lib* forage (tailored to individual)<br>Top quality forage sourced from anywhere in the world and feed<br>Gd quality feed to individual's needs and gd dust free forage<br>'Hay gain' to steam hay to minimise dust<br>High standard of forage<br>Unlimited high quality haylage/hay<br>Feed, liberal roughage, *ad-lib* hay, hard feed based on current exercise<br>Specialist feed (tailored to each horse)
**Number of times fed (5 statements)**	**Number of times fed (3 statements)**
No variety in [rigid] routine of feeding<br>One feed 05.00<br>One feed 12.00<br>One feed 18.00<br>Hard feed am and pm. Hay/haylage twice a day<br>Forage twice a day, concentrates 2/3 times<br>Hard feed 10%, forage at night<br>Severely restricted forage and too much concs proportionately	Feed 4/3 x day. Gd quality food, optimum amounts to maintain weight. Balance for exercise<br>Minimum 3xdaily regular balanced diet and appropriate formula for work done<br>*Ad-lib* forage monitored by staff

Water (4 statements)	Water (2 statements)
Ad- lib clean water (4 x)	*Ad-lib* clean water<br>Automatic water

Table S5. Stakeholder perceptions of minimum and best 'life' with regard to daily routine and monitoring (26 statements, ranked 5th).

Minimum standards (13 statements in total)	'Best life' (13 statements in total)
**Routine (7 statements)**	**Routine (5 statements)**
Routine<br>Daily check<br>Routine<br>Routine<br>Changing routines affect welfare<br>No change of scenery the same route to the gallops and back day in, day out<br>Limited routine, up one gallop every day	Established routine<br>Change routine if it's not working<br>Routine/variety, feed/exercise/staff<br>Individual work routine and care<br>Great ridden routine, turn out, swimming (tailored to each horse)
**Care (3 statements)**	**Care (4 statements)**
Don't get routine care at morning and night, everyone too busy<br>Visual contact of horse<br>Relaxation time in box limited	Attention to detail when looking after the horse daily<br>Limiting [dangerous] factors in day to day life<br>Pick of grass if no turnout<br>Getting comfortable with new/unusual experiences (e.g. racing) tailored to suit the horse
**Horse-human relationship (2 statements)**	**Horse-human relationship (3 statements)**
Treating all horses in the same way so one size fits all<br>Minimal staff and time spent with horse	Grooming daily<br>Consistency of routine and carer<br>Same person looking after and riding the same horse every day if they get on
**Staff ratio** 6 horses:1 lad **(1 statement)**	**Good horse: staff ratio** 3 horses:1 lad **(1 statement)**

Table S6. Perceptions of participants' opinions regarding minimum and best 'life' with regard to policy and procedures (16 statements, ranked 6th). There were no minimum welfare standards statements.

Standard procedures (8 statements in total)	Policy 'Best life' (8 statements in total)

Assessment of welfare using behavioural and physiological methods indicates an animal can perform at the top level with good welfare<br>Trainers monitors health of horses in a systematic way<br>On-going individual assessment and programme of work and care for horses<br>Daily vet care, monitoring system/historical data collected<br>Where trainers have undergone competence based training rather than tick box, attend all modules<br>Checking yards where staff are always leaving<br>Rider coaching and development, make them better	An agreed set of interventions for racing clearance post stand downs (where there is a set protocol to follow)<br>Some assessment of horses at the races that covered pre and post-race management, which included warming up and cooling down.<br>Lameness routine, assessment at racecourse prior to racing<br>Lameness, gold standard investigation and treatment if needed<br>Standard set of criteria for wind ops and surgery with a set stand down [period] before running again<br>CPD for trainers, would have a measurable impact on welfare<br>Regulatory framework for manage welfare issues<br>Post race life is accounted for
**Drug and veterinary intervention (1 statement)**	**Drug and veterinary intervention (No statements)**
Therapeutic use exemptions (TUE's) omezaprazole, regumate, [the restrictions placed on the administration of some drugs before racing needs addressing	

Table S7. Stakeholder's perceptions of minimal welfare standards and 'best life' with regard to turnout/social contact (16 statements, ranked 7th).

Minimum welfare standards (4 statements in total)	'Best life' (12 statements in total)
**Turnout (2 statements)**	**Turnout (8 statements)**
Degree of turnout for same period each day, with/without grazing<br>Turnout whatever the weather conditions, even when it's too hot and no care	Turn out every every day, elements, space/grass/outdoors/being able to buck and kick<br>Paddock living with access to shelter by choice<br>Access to grass-may be controlled<br>Choice of access to outside space<br>Keeping horses' lives as natural as possible<br>Daily access (weather permitting) to turnout<br>Paddock turnout every day dependant on weather<br>Availability of turn out paddocks
**Social contact (1 statement)**	**Social contact (4 statements)**
Visual access to other horses	Wider access to social companions, group turnout (appropriately managed)<br>Adjoining paddocks (non-damaging biosecurity)<br>Adjoining paddocks, groom but not kick<br>Adequate mental stimulation
**Human-horse relationship (1 statement)**	**Horse-human relationship (0)**
Same lad/lass looking after horse with	

regard to turnout

Table S8. Perceptions of participants' opinions regarding minimum and best 'life' with regard to staff management and education (15 statements, ranked 8th).

Minimum welfare standards (6 statements in total)	'Best life' (9 statements in total)
**Care (5 statements)**	**Care (8 statements)**
Staff not consistent and don't notice if the horse is unwell or off colour<br>Caring and capable staff<br>Competent knowledgeable staff/trainer<br>More education of those involved in racing<br>Care, no abuse from staff	Good staff, kind, care and awareness and<br>experienced rider ability<br>Experienced knowledgeable staff who will listen<br>Knowledgeable empathetic staff<br>Skilled staff in handling and riding<br>Experienced staff who know if a horse is lame<br>Well trained staff<br>Caring well trained staff<br>Knowledgeable experienced staff
**Management (1)**	**Management (1)**
Understanding of racing/training education rather than licence through 'who you know, how much money you have'	Strong leadership and management in the yard

Table S9. Perceived challenges to racehorse welfare

Participants were asked to record, on sticky notes, the perceived challenges the racing industry needed to address in terms of racehorse welfare. In total participants provided 68 statements which after analysis were placed in one of the eight themes, as illustrated below.

1.Health/veterinary care (14 statements, ranked 1st)

Soundness
On-going health issues-repetitive injuries
Medication of joints.
Chronic disease monitoring including dentistry, veterinary aspects, pain management more important than management issues.
Pain (injury/ulcers)
Revisit the use of medicines not allowed to give after racing, e.g., omeprenazole
[The need for] greater robustness of substance analysis
Overuse of veterinary interventions
Surgery for no suitable reason
Monitor welfare at sales/horses too buzzed up/too many scopes
Speed kills/injury
Travel without hay (some no hay 24 hrs before racing)
Breathlessness
Wind ops

2. Staff education and education (12 statements, ranked 2nd)

[Lack of} quality staff and trainers
[Lack of} good quality staff
Lack of experienced staff

[Lack of} sufficient well trained staff
[Not] enough staff with experience and knowledge
[Lack of] basic knowledgeable staff
Bad trainers
Bad riders/staff
Competent proprietors
Trainers education – need a trainer's exam
Trainers not listening to lads (re lameness)
BHA should monitor trainers who are always sending back stable passes and the BHA should investigate them

3. Daily routine and monitoring (8 statements, ranked 3rd)

Having time to spend exercising and on routine exercising
Little relaxation time for horses in yards
Daily routine of food and work of horses
Current routine in flat yards (*starting so early all the year round)*
Staff/horse ratio
Staff and trainers to know their horses
The standard of care in terms of horse husbandry, stable routine, 'looking round.'
Too much poor racing and a lack of staff to look after the horses back at the yard as people are off racing

4. Training/exercise and recovery (8 statements, ranked 4th)

Rushed recovery after racing
Horses labelled as ungenuine when their race day experience all rushed, still blowing when on the lorry
No recovery time after race
Overtraining, no time to adapt
Starting horses too young
Travelling horses in hot weather
Time- horses rushed when broken in
Sales- breeze ups

(Some of the suggestions that were put forward in the final task, 'New practices they have seen' could be interpreted as challenges for the industry to deal with. These include: - Breeze ups putting too much pressure on young mentally and physically immature horses. Horses broken in properly, so they have another career as well as making them easier to deal with).

5.Physical comfort/living (6 statements, ranked 5th)

Cleanliness of establishment
Standard of stables, ambience
Level of health in yard (in terms of yard hygiene, flu, herpes, low grade bacterial infection)
'Stable' environment for horses
Good up to standard facilities, paddocks, boxes, gallops
Variety of saddle options

6. Turnout/social contact (6 statements, ranked 6th)

Meeting of mental needs, social behaviour
Mental attitude and happiness
Being able to express normal behaviour/ socialise
Ability to build personal relationship with lad
Lack of turnout and social companionship with other horses
Mental health

7. **Policy and procedures (4 statements, ranked 7th)**

Owners grabbing horses after winning (Health and Safety)
BHA should be split- governance and regulation
Owners driving handicapping system
Running over unsuitable trips

8. Other areas (3 statements, ranked 8th)
Being owned by an owner who doesn't understand racing
Breeding for performance rather than breeding from 'tough' horses that had proved themselves without lots of veterinary intervention
That public perception does not match with actual welfare issues
Value of horses after their racing career has finished

9. Feeding (2 statements, ranked 9th)

Enough relevant feeding
Basic feeding to prevent ulcers and digestive issues
